# Chromosomal-Scale Genome Assemblies of Two Coastal Plant Species, *Scaevola taccada* and *S. hainanensis*—Insight into Adaptation Outside of the Common Range

**DOI:** 10.3390/ijms24087355

**Published:** 2023-04-16

**Authors:** Sen Li, Xiaomeng Mao, Ziwen He, Shaohua Xu, Zixiao Guo, Suhua Shi

**Affiliations:** 1 State Key Laboratory of Biocontrol, Guangdong Provincial Key Laboratory of Plant Resources, School of Life Sciences, Sun Yat-sen University, Guangzhou 510275, China; 2 Department of Ecology and Genetics, Plant Ecology and Evolution, Uppsala University, Norbyvägen 18D, 75267 Uppsala, Sweden; 3 School of Ecology, Sun Yat-sen University, Guangzhou 510275, China; 4 Southern Marine Science and Engineering Guangdong Laboratory (Zhuhai), Zhuhai 519082, China

**Keywords:** genome assembly, *Scaevola taccada*, *Scaevola hainanensis*, evolution, adaptation, *FAR1*

## Abstract

While most of the species in Goodeniaceae family, excluding the *Scaevola* genus, are endemic to Australasia, *S. taccada* and *S. hainanensis* have expanded their distribution range to the tropical coastlines of the Atlantic and Indian Oceans. *S. taccada* appears to be highly adapted to coastal sandy lands and cliffs, and it has become invasive in places. *S. hainanensis* is found mainly in salt marshes near mangrove forests, and is at risk of extinction. These two species provide a good system to investigate adaptive evolution outside the common distribution range of this taxonomic group. Here, we report their chromosomal-scale genome assemblies with the objective of probing their genomic mechanisms related to divergent adaptation after leaving Australasia. The scaffolds were assembled into eight chromosome-scale pseudomolecules, which covered 90.12% and 89.46% of the whole genome assembly for *S. taccada* and *S. hainanensis*, respectively. Interestingly, unlike many mangroves, neither species has undergone whole-genome duplication. We show that private genes, specifically copy-number expanded genes are essential for stress response, photosynthesis, and carbon fixation. The gene families that are expanded in *S. hainanensis* and contracted in *S. taccada* might have facilitated adaptation to high salinity in *S. hainanensis*. Moreover, the genes under positive selection in *S. hainanensis* have contributed to its response to stress and its tolerance of flooding and anoxic environments. In contrast, compared with *S. hainanensis*, the more drastic copy number expansion of *FAR1* genes in *S. taccada* might have facilitated its adaptation to the stronger light radiation present in sandy coastal lands. In conclusion, our study of the chromosomal-scale genomes of *S. taccada* and *S. hainanensis* provides novel insights into their genomic evolution after leaving Australasia.

## 1. Introduction

The family Goodeniaceae comprises approximately 400 species classified into twelve genera [1]. Excepting the *Scaevola* genus [2], the other eleven genera are almost all endemic to Australasia [3]. *Scaevola taccada* (Gaertner) Roxburgh (*Scaevola sericea* Vahl is a synonym of this species) and *Scaevola hainanensis* Hance are two *Scaevola* species distributed outside Australasia. *Scaevola* species vary considerably in floral morphology, fruit morph, and habitat, and their plant types vary from herbs, scramblers, and shrubs to small trees [2,3].

*S. taccada* is widespread through the Pacific and Indian Oceans, inhabiting almost the whole global tropical and subtropical coastline [2,4]. *S. taccada* was introduced to the Americas for ornamental use, with invasion and colonization in tropical areas of Mexico being reported in recent years [5]. *S. taccada* produces dimorphic fruits, with one type dispersing by ocean current (with cork and pulp, floating > 180 days in seawater) and the other type by birds (with only pulp and less float, preferred by frugivorous birds) [6]. This leads to its colonization capability and radiation speciation [2,5]. *S. taccada* is an erect shrub or small tree that can be as tall as 7 m. It prefers open coastal sands or rocks exposed to direct sunlight and salty air, forcing it to regulate photosynthesis. Goldstein et al. [7] found that the growth and photosynthetic response of *S. taccada* could maintain a positive carbon balance to adapt to the dynamic, high-salinity environment. *S. taccada* has been widely used in coastal landscaping for preventing coastal erosion, soil reclamation, and ornamentation [5].

*S. hainanensis* is only found in mangrove forests and salt marshes, with a narrow distribution along the coasts of South China and Vietnam [8]. *S. hainanensis* is a creeping fleshy shrub with 1–2.5 cm × 0.2–0.5 cm spatulate leaves [8,9]. It is at high extinction risk due to frequent artificial disturbance. It is recorded as an endangered species in South China (including Taiwan province) [10,11]. *S. hainanensis* has adapted to tolerate high salinity and strong UV in intertidal habitats, which are extreme for woody plants.

Although both S. *taccada* and *S. hainanensis* have distribution ranges out of Australasia, *S. taccada* is widely distributed and even invasive in places, while *S. hainanensis* is narrowly distributed and at extinction risk. Moreover, they have each adapted to different habitats. Hence, these two species provide a good model to study adaptive evolution to new environments outside the common range.

An important difference between the coastal sandy beach habitat of *S. taccada* and the intertidal habitat of *S. hainanensis* is light intensity. Light is one of the most important environmental factors; it participates in multifaceted growth and development processes, including seed germination, hypocotyl growth, chlorophyll synthesis, stomata opening, and flower initiation [12,13,14,15]. *S. taccada* trees are tall, bearing much stronger light, especially UV light, while the short *S. hainanensis* shrubs are usually sheltered by mangrove forests. The five phytochrome genes (*PHYA*-*PHYE*) encode phytochrome photoreceptors that regulate various responses to red and far-red light. PHYA and PHYB are the two most abundant phytochromes, and PHYA is the primary photoreceptor for mediating far-red light signaling [16]. *FAR1* genes are essential for PHYA signaling, and play important roles in diverse physiological and developmental processes, such as UV-B signaling, circadian clock entrainment, flowering, chlorophyll biosynthesis, and ABA signaling [13,16,17,18,19]. It remains unknow how these gene families have evolved in the two *Scaevola* species.

The unavailability of genome sequences is a hinderance to investigation of these questions. Therefore, we sequenced and assembled the chromosome-level genome assemblies of *S. taccada* and *S. hainanensis*. In addition, we conducted comparative genomic analyses to clarify the genomic mechanisms of environmental adaptation of the two species. These two high-quality genomes can provide a valuable resource for future research on trait improvement, artificial breeding, and conservation.

## 2. Results

### 2.1. Genome Assembly and Annotation

**Figure 1:**
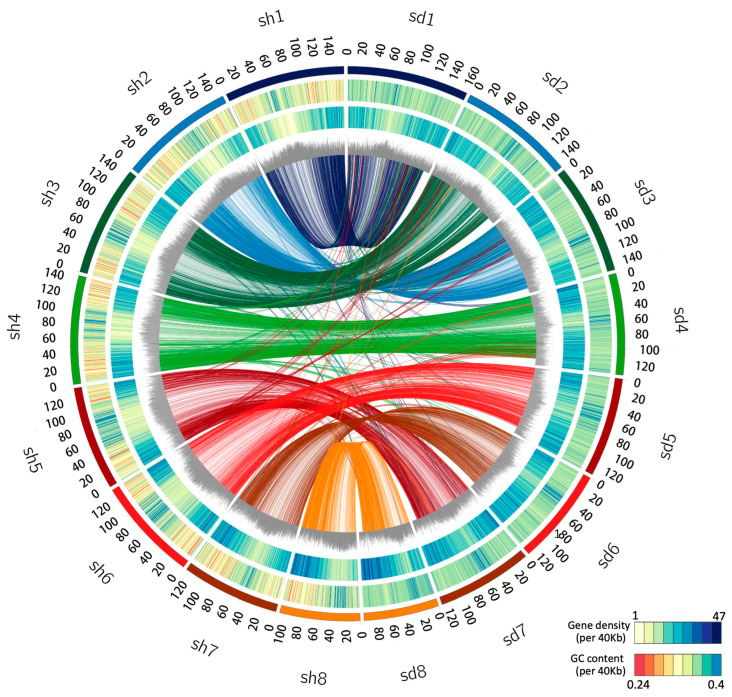
Genomic landscape of the *S. taccada* and *S. hainanensis* assemblies. Circos view of the *S. taccada* (sd, (**right side**)) and *S. hainanensis* (sh, (**left side**)) genome assemblies. From the outer to the inner, the circular tracks represent pseudochromosomes, GC content (0.24–0.4 per 40 kb), gene density (1–47 per 40 kb), percentage of repeats (0.3–0.9 per 40 kb), and collinear links. Each line in the circle’s center connects a pair of homologous genes between *S. taccada* and *S. hainanensis*.

We combined the 10× genomics paired-end sequencing reads, paired-end short reads, and Hi-C (chromosome conformation capture) sequence data to de novo assemble the genomes of *S. taccada* and *S. hainanensis*. After assembly, correction, and polishing, the sequences were assembled into eight pseudo-chromosomes corresponding to their karyotype of 2*n* = 16. The final genome assembly of *S. taccada* was 1.11 Gb in length, with the pseudo-chromosomes ranging from 92 Mb to 150 Mb. The final genome assembly of *S. hainanensis* was 1.24 Gb in length, with the pseudo-chromosomes ranging from 106 Mb to 159 Mb (Table 1, Figure 1). The sizes of these genome assemblies are larger than the estimated genome sizes from Genome Scope, though smaller than the values from Genome Character Estimator (Table S1 in supporting information). The pseudo-chromosomes covered 90.12% and 89.46% of the whole genome assembly for *S. taccada* and *S. hainanensis*, respectively, indicating a chromosomal-level assembling continuity.

We examined the assembly completeness by aligning paired-end short reads to the assemblies [20]. In total. 97.37% and 99.29% of the short reads were successfully mapped to the genome assemblies of *S. taccada* and *S. hainanensis*, respectively. Using Benchmarking Universal Single-Copy Orthologues (BUSCO) [21], 94.5% and 93.8% of the embryophyta core genes (the embryophyta_odb10 database) were identified as complete (single copy or duplicated) (Table 1). The high proportions of complete BUSCOs support the completeness of our assemblies.

Using a combination of homology-based search and de novo prediction, we estimated that 61.25% and 69.76% of the *S. taccada* and *S. hainanensis* genome assemblies respectively consist of repetitive sequences (see Table S2 in the supporting information). With repetitive elements masked, 34,560 and 34,033 gene models were predicted and 30,016 (86.85%) and 31,136 (91.49%) gene models were functionally annotated in the *S. taccada* and *S. hainanensis* genomes, respectively (Table S3).

### 2.2. Whole Genome Duplication and LTR Retrotransposon Insertion

**Figure 2:**
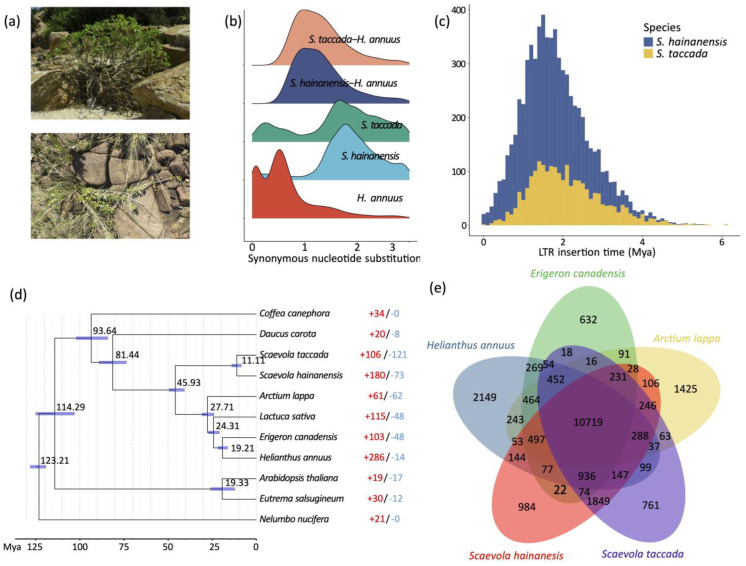
Gene family and LTR-RT evolution. (**a**) Photos of *S. taccada* (upper) and *S. hainanensis* (lower) in Hainan Island, China. (**b**) Ks distribution between intraspecific paralogous genes and interspecific homologous genes. (**c**) LTR insertion time. (**d**) Phylogenetic tree of *S. taccada*, *S. hainanensis* and nine related species. The estimated divergence time is shown beside each node. Blue bars are 95% confidence intervals. Red and blue numbers indicate the numbers of significantly expanded and contracted gene groups. (**e**) Shared and private gene groups in *S. taccada*, *S. hainanensis* and relatives *Helianthus annuus*, *Arctium lappa*, and *Erigeron canadensis*.

Whole-genome duplication (WGD) events and transposon insertions are important drivers of plant genome size expansion. We first sought evolutionary relics of WGD in *S. taccada* and *S. hainanensis* genomes by detecting paralogous synteny gene blocks based on all-to-all blastp alignments [22]. However, we only identified 1382 (4.00%) syntenic genes from 85 collinear blocks in the *S. taccada* genome and 1302 syntenic genes (3.83%) from 73 collinear blocks in the *S. hainanensis* genome (Figure 1). The peak of synonymous nucleotide substitutions (*Ks*) of syntenic gene pairs corresponds to the paleohexaploidisation (γ-WGD) in the ancestors of all extant eudicots (Figure 2b). These results agree with the previous view that the Goodeniaceae lineage has not experienced a WGD event after the γ-WGD event [23].

Long-terminal-repeat (LTR) retrotransposons were the predominant component among all repetitive elements, taking percentages of 57.13% and 42.44%, respectively (Table S2). Notably, the relatively smaller numbers of LTR retrotransposons and the larger number of unclassified transposons in the *S. hainanensis* genome may be due its genome assembly being more fragmented. We calculated the insertion times of LTR retrotransposon elements by comparing the 5′ end and 3′ end LTR sequences. The insertion of LTR retrotransposons started as early as six million years ago and increased significantly to the peak 1–2 million years ago (Figure 2c). This rapid insertion resulted in a large number of LTR retrotransposons reserved in the *S. taccada* and *S. hainanensis* genomes. Hence, we infer that LTR-RT insertion has likely contributed to the relatively large genome size of the two species and to adaptive evolution in their invasion of new distribution ranges outside of Australasia.

### 2.3. Phylogeny Construction and Gene Copy Number Evolution

We first clustered the genes of *S. taccada*, *S. hainanensis*, and nine other angiosperm species (*Helianthus annuus*, *Lactuca sativa*, *Daucus carota*, *Arctium lappa*, *Erigeron canadensis*, *Coffea canephora*, *Arabidopsis thaliana*, *Eutrema salsugineum*, and *Nelumbo nucifera*) into homologous gene groups, with 31,225 groups identified. Among these gene groups, 8266 were shared by all species and 658 were single-copy orthologous groups. In order to date the divergence between *S. taccada* and *S. hainanensis*, the 658 single-copy genes were used for phylogenetic tree construction. With the constructed phylogeny, we estimated that *S. taccada* and *S. hainanensis* diverged ~11 million years ago (Figure 2d).

We found 572 gene families containing 5771 genes and 703 gene families containing 4192 genes that were private to *S. taccada* and *S. hainanensis*, respectively (Figure 2e). The functional enrichment analysis showed that both the *S. taccada* and *S. hainanensis* genes were significantly overrepresented by gene ontology (GO) terms related to stress response, such as oxidoreductase activity (GO:0016702), heat shock protein (K03283), and serine/threonine-protein kinase (K20718 in *S. taccada* and K17545 in *S. hainanensis*). The genes private to *S. taccada* were significantly enriched in the process of photosynthesis (GO:0009767, GO:0009521) and transmembrane protein (K20724, K17086). These genes might have contributed to the growth and photosynthetic response (Tables S4 and S5). The genes private to *S. hainanensis* were significantly enriched in the carbohydrate metabolic process (GO:0005975), which might have contributed to nutrient utilization.

We identified 106 gene groups that were significantly expanded and 121 gene groups that were significantly contracted in *S. taccada* (Figure 2d). Genes in the expanded gene groups of *S. taccada* were significantly enriched in the processes of photosynthesis (GO:0009521, GO:0009767, GO:0009772, GO:0019684, and K02704), DNA repair (GO:0006281), oxidoreductase activity (GO:0016705), and carbohydrate metabolism (GO:0005975) (Tables S6 and S7). In *S. hainanensis*, 180 gene groups were significantly expanded and 73 gene groups were significantly contracted (Figure 2d). Genes in the expanded gene families of *S. hainanensis* were significantly enriched in the processes of protein serine/threonine kinase activity (GO:0004674, K17535, K13428, K02178), photosynthesis (GO:0015979, GO:0009522), carbon fixation (GO:0015977), response to stress (GO:0031347, GO:0006950), and disease resistance (K13459, K13457).

**Figure 3:**
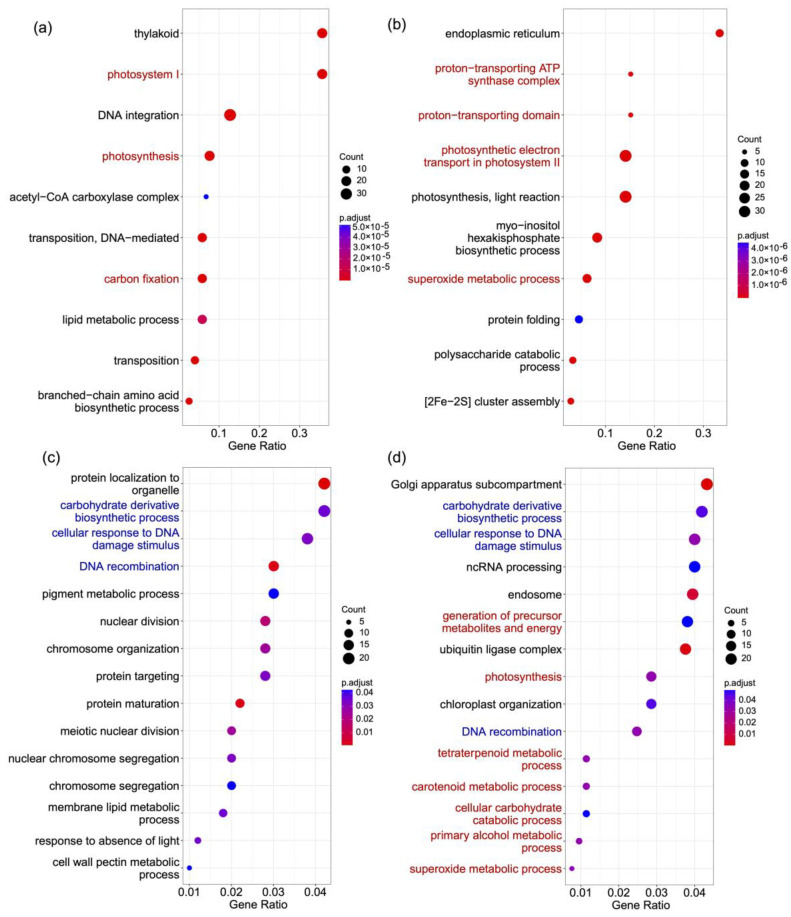
GO terms overrepresented in different gene lists. (**a**) Genes in families significantly expanded in *S. taccada* and contracted in *S. hainanensis*. (**b**) Genes in families significantly expanded in *S. hainanensis* and contracted in *S. taccada.* (**c**) Genes under positive selection in *S. taccada.* (**d**) Genes under positive selection in *S. hainanensis.* GO terms shared by (**c**,**d**) are highlighted in blue. GO terms indicating differential adaptation of the two species are highlighted in red.

We found 62 gene families that are significantly expanded in *S. hainanensis* and significantly contracted in *S. taccada*. Genes in these families were overrepresented by GO terms related to proton and electron transport and to superoxide metabolism (Table S8, Figure 3a,b). The expansion of genes involved in proton and electron transport in *S. hainanensis* may contribute to its tolerance of high salinity in mangrove habitats. In contrast, 92 gene families are significantly expanded in *S. taccada* and significantly contracted in *S. hainanensis*. Genes in these families were significantly overrepresented by GO terms related to photosynthesis and carbon fixation (Table S8, Figure 3a,b). The copy number increase of genes involved in photosynthesis and carbon fixation is consistent with *S. taccada* growing to form tall trees in coastal sand and rock environments.

### 2.4. Genes under Positive Selection

We used the “branch-site model” in PAML v4.8 [17] to detect the genes under positive selection in *S. taccada* and *S. hainanensis* from a set of 4317 high-confidence orthologs. We found 515 (Table S9) and 542 (Table S10) genes under positive selection in the *S. taccada* and *S. hainanensis* lineage, respectively. The GO terms carbohydrate derivative biosynthetic process, cellular response to DNA damage stimulus, and DNA recombination are overrepresented in both lists of genes under positive selection (Tables S11 and S12, Figure 3c,d). However, GO terms related to metabolic processes, including carotenoid, alcohol, and superoxide metabolism, are only overrepresented in the genes under positive selection in *S. hainanensis* (Table S12, Figure 3c,d). These metabolism-related genes are likely important for *S. hainanensis* to respond to stress and to tolerate flooding and anoxic environments.

Particularly, certain genes are under positive selection on the common ancestor branch of the two species (Table S13). Nine genes that are under positive selection have functions related to light sensitivity and regulation (Table S14). These nine genes might contribute to the response to intense light stress, photosynthesis, and carbon fixation. For examples, *XCT* and *APRR5* are circadian-associated. *XCT* encodes the XAP5 family protein, which is involved in light regulation of the circadian clock and photomorphogenesis. *APRR5* encodes a pseudo-response regulator with mutations that affect various circadian-associated biological events.

Another 18 genes are involved in response to multiple stresses (Table S14). For example, three genes likely have functions in salt tolerance; *VOZ1* positively regulates several salt-responsive genes, *SIA1* is an atypical protein kinase induced by salt stress, and *DELTA-OAT* encodes an ornithine delta-aminotransferase, which responds to salt stress.

### 2.5. Evolution of the FAR1 Gene Family in S. taccada and S. hainanensis

**Figure 4:**
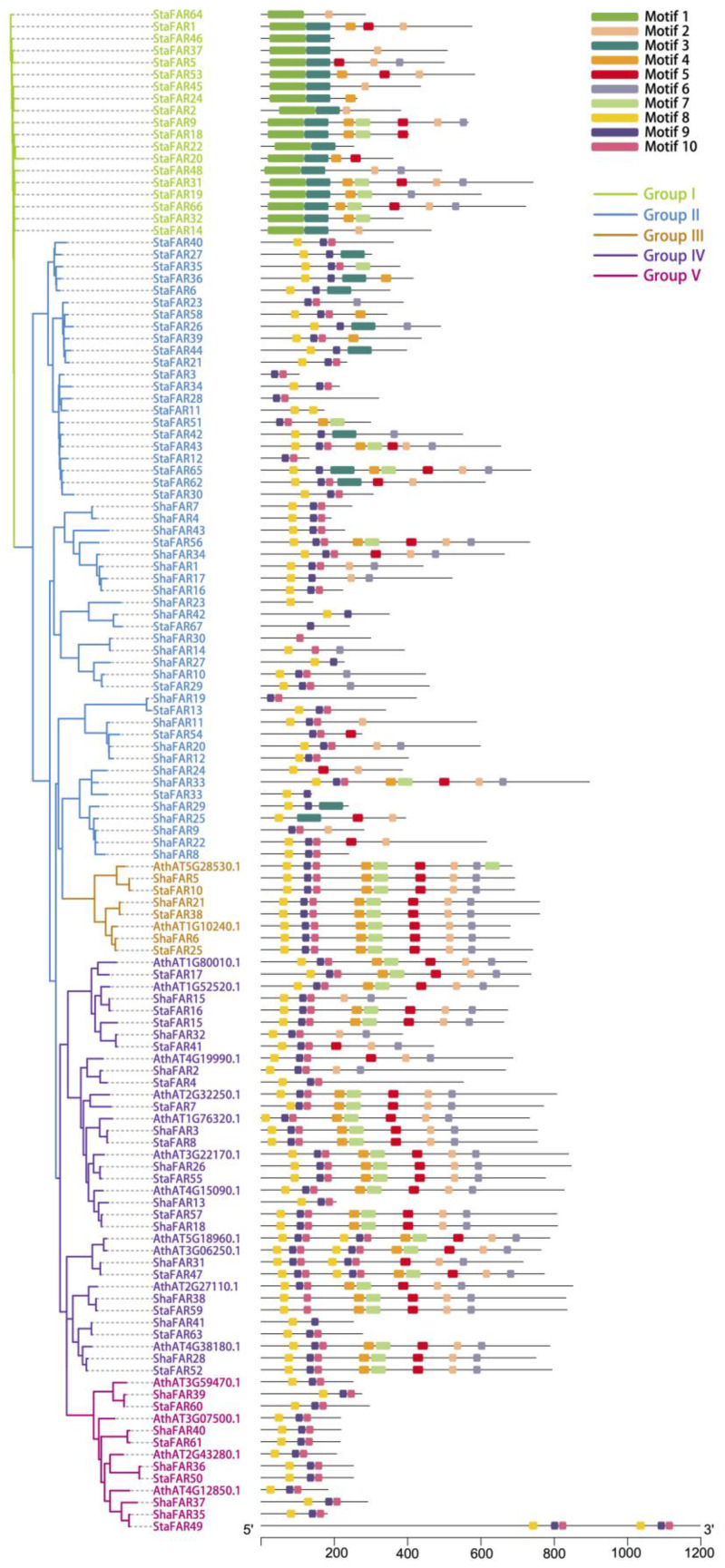
Phylogenetic tree of *FAR1* genes in *S. taccada*, *S. hainanensis* and *A. thaliana*. The prefixes “Sta”, “Sha”, and “Ath” stand for *S. taccada*, *S. hainanensis*, and *A. thaliana*, respectively. Motifs predicted for each gene are shown on the right.

In both *S. taccada* and *S. hainanensis*, we found only four genes that are homologous to the *PHYA*-*PHYE* genes. That is to say, there is no copy number differentiation in phytochrome genes between the two species. In comparison, we identified 67 and 43 genes belonging to the *FAR1* family in the genomes of *S. taccada* and *S. hainanensis*, respectively. These genes were named *StaFAR1*–*StaFAR67* and *ShaFAR1*-*ShaFAR43* following their chromosomal positions (Tables S15 and S16). The conserved domain motifs of *FAR1* members were identified (Figure 4).

**Figure 5:**
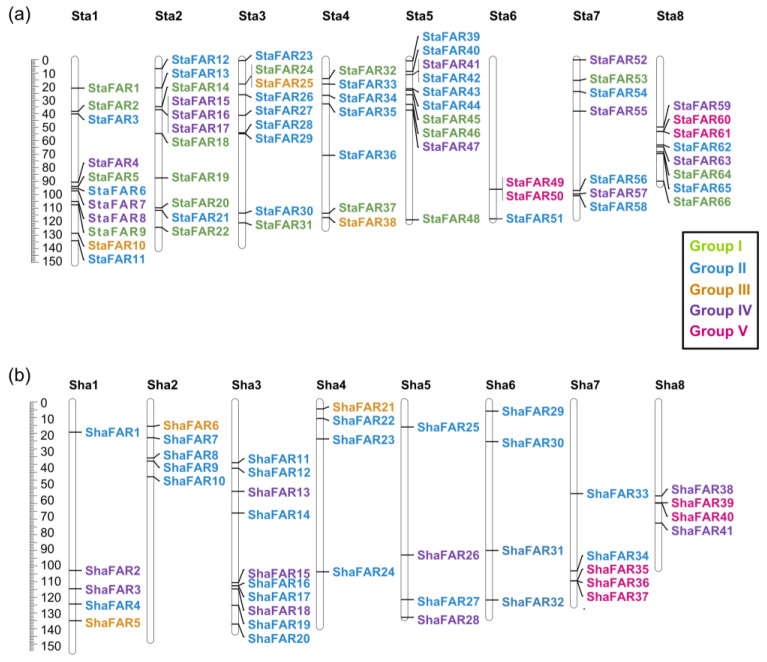
Chromosomal locations of the *FAR1* genes on the eight chromosomes of (**a**) *S. taccada* and (**b**) *S. hainanensis*. Sta: chromosomes of *S. taccada*. Sha: chromosomes of *S. hainanensis*.

We aligned the *FAR1* protein sequences of *S. taccada*, *S. hainanensis*, and *A. thaliana* to construct a phylogenetic tree using the maximum likelihood method. According to their phylogenetic relationship and motif pattern, these *FAR1* genes were divided into five groups: Group I comprises 19 *FAR1* members from *S. taccada*; Group II contains 28 and 24 *FAR1* members from *S. taccada* and *S. hainanensis*, respectively; Group III contains 3, 3, and 2; Group IV contains 13, 11, and 11; and Group V contains 4, 5, and 4 members from *S. taccada*, *S. hainanensis*, and *A. thaliana*, respectively (Figure 4). We found 66 and 41 of these *FAR1* genes located on the chromosome-anchored scaffolds in *S. taccada* and *S. hainanensis*, respectively. Moreover, these genes are evenly distributed on the chromosomes (Figure 5).

We inferred how the *FAR1* gene family has increased their copy number, revealing two pairs of tandem duplication and 17 pairs of transposed duplication in *S. taccada* (Table S17). In comparison, one pair was tandem duplicated, two pairs were proximally duplicated, and six pairs were transposed in *S. hainanensis* (Table S18). In short, transposed and tandem duplication have played a key role in *FAR1* gene family expansion.

## 3. Discussion

The family Goodeniaceae has recently experienced evolutionary radiation, leading to the current species diversity of about 400 species. The *Scaevola* genus consists of ~130 species worldwide, with the center of origin and distribution being Australasia. There are about 40 *Scaevola* species that are distributed outside Australasia. Both *S. taccada* and *S. plumieri* are widespread across tropical regions, while other species are scattered in tropical islands. *S. hainanensis* is distributed in the coastal areas from southern China to northern Vietnam. *S. taccada* is the only *Saevola* species with a distribution overlapping with *S. hainanensis.*

Polyploidy and transposon insertions are two important drivers of genome evolution, providing genomic materials for evolutionary innovation [24,25,26]. Whole-genome duplication events have been demonstrated to contribute to species divergence and morphological diversification in Asterids [23]. A combination of *Ks*-based and synteny analyses showed that *S. taccada* and *S. hainanensis* have experienced no recent WGD events after the well-known paleohexaploidisation (γ-WGD) event. However, their genomes consist of large proportions of LTR-RTs which were inserted into the genomes within the past several million years. These recent and rapid LTR-RT insertions might have been the main driver of genomic evolution which helped these species to move out of Australasia.

Plants growing in coastal regions are often subject to UV, high salt stress, and periodic tides. The different rates of gene gain and loss usually cause the copy number of homologous gene groups to vary among species, which provides the genetic basis for phenotypic innovation, species diversification, and genome size evolution [27,28]. Both the genes private to and the gene groups expanded in *S. taccada* and *S. hainanensis* are enriched in the GO and KEGG terms related to photosynthetic, heat shock protein, and serine/threonine-protein kinase. Hence, gene copy evolution might have contributed to abiotic stress signaling and responses in these two species.

We found that genes under positive selection have contributed to the maintenance of photosynthesis, response to stress, and regulation of the circadian clock, which is important for *S. taccada* and *S. hainanensis* to adjust to the light and tidal rhythm. We concluded that these private genes, copy-number expanded genes, and positively-selected genes are essential for the two species to adapt to their extant habitats.

We identified both *S. taccada* and *S. hainanensis* as having more *FAR1* gene copies than closely related species (7 in *Daucus carota*, 1 in *Helianthus annuus*, 10 in *Lactuca sativa*, and 24 in *Solanum pennellii*; data from PlantTFDB [29]). Tandem duplication and transposed duplications have made the key contribution to the copy number expansion of *FAR1* genes in these two species. In particular, *S. taccada* has more *FAR1* genes than *S. hainanensis*. The larger number of *FAR1* genes in *S. taccada* may be beneficial because the *S. taccada* trees are more likely to be injured by strong light in their coastal sandy land habitats as compared with the mangrove understory habitat of *S. hainanensis*.

In conclusion, we have reported the first chromosome-level genome assemblies of *S. taccada* and *S. hainanensis.* By comparative genomic analysis, we have revealed the mechanisms underlying their adaptation outside of their common range in Australasia. Our genomic analyses provide insights into their adaptation to different environments in intertidal zones and coastal sands. To the best of our knowledge, the two genome assemblies reported here are the first available public genome resource on the Goodeniaceae family. Our genome research provides a reference for studies of the invasion ecology of *S. taccada* and conservation ecology of *S. hainanensis.* Moreover, our genome resource can benefit subsequent studies on genetic diversity, demographic history, radiogenic evolution, hybridization, and biogeographic processes in these species.

## 4. Material and Methods

### 4.1. Plant Material

The *Scaevola taccada* and *S. hainanensis* materials used for genome sequencing were collected from Dongzhai Harbor (110°35′35.79 E, 19°55′10.05 N) and Daoxue village (110°37′01.31 E, 19°57′47.38 N), Haikou, Hainan province, China, respectively. Total genomic DNA was extracted from leaves following the CTAB method [30]. The genomic DNA sample was quantified using a Nanodrop 2000 Spectrophotometer (NanoDrop Technologies, Wilmington, DE, USA) and assessed by 1% agarose gel electrophoresis. To assist genome annotation, RNA was extracted from the flowers and leaves of *S. hainanensis* and the flowers, fruit, and leaves of *S. taccada* following the modified CTAB method [31]. These genomes were sequenced as part of a project aiming to sequence mangrove species worldwide [32].

### 4.2. 10× Genomics Sequencing

Only reads over 50 Kb long were retained for 10× genomics library construction. The prepared 10× genomics library was then sequenced as 150 bp paired-end reads on a BGISEQ-500 platform. Data quality control was performed using SOAPnuke v 2.7.1 [33] software, yielding c. 109.06 Gb of bases for *S. taccada* and c. 81.48 Gb of bases for *S. hainanensis*, respectively.

### 4.3. Hi-C Sequencing

Tender leaves were collected for Hi-C libraries construction. The leaves were fixed with formaldehyde and lysed and the cross-linked DNA was digested with MboI. Then, 100-bp paired-end Hi-C libraries were constructed and sequenced on a BGISEQ-500 platform. After removing low-quality reads with SOAPnuke v2.7.1 software, we yielded c. 125.54 Gb and 132.63 Gb of bases for *S. taccada* and *S. hainanensis*, respectively.

### 4.4. Short-Read Sequencing

DNA short-read sequencing was performed for gap-filling and error correction. Purified DNA was used for 150-bp paired-end libraries construction and then sequenced on a BGISEQ-500 platform. After quality control by SOAPnuke v2.7.1, we yielded c. 84.52 Gb and 103.02 Gb of bases for *S. taccada* and *S. hainanensis*, respectively.

The RNA-seq libraries were sequenced on a BGISEQ-500 platform for gene prediction. For *S. taccada*, we yielded c. 5.59 Gb, 5.86 Gb, and 6.64 Gb of bases from sequencing of flowers, fruits, and leaves, respectively. For *S. hainanensis*, we yielded c. 6.96 Gb sequences from sequencing of flowers and c. 11.67 Gb from leaves.

### 4.5. De Novo Genome Assembling

Before assembling, we used the short reads to estimate genome size using 17-mer of jellyfish v2.2.10 [34] and genomic character estimator (GCE) v1.0.2 [35]. The quality-filtered reads were then used for genome assembling. We first assembled the *S. taccada* and *S. hainanensis* genomes based on the 10× reads using Supernova v2.0.1 [36]. The draft assembly was corrected and gap-filled based on paired-end short reads using GapCloser v1.2.1 [37]. Hi-C reads were qualified to improve the assemblies using HiC-Pro v3.2 [38] and then mapped to the polished 10× genomes to obtain valid reads with contact information. The Hi-C maps were generated by Juicer v3 [39]. The scaffolds were visualized and manually adjusted by Juicebox v1.8.0 [40] and anchored to the chromosome level using the 3D de novo genome assembly (3D-DNA) pipeline [41].

### 4.6. Genome Annotation

Repetitive elements within the *S. taccada* and *S. hainanensis* genome assemblies were identified using combined homology-based and de novo-based approaches. The whole genome sequences were aligned with RepBase [42] using the RepeatMasker v4.1.2 program [43] to identify the known transposable elements. The de novo repeat libraries were constructed using RepeatModeler v2.0.2 [43]. The unknown transposon sequences were classified using TEclass v2.1.3c [44]. In addition, tandem repeats were identified by the TANDEM REPEAT FINDER v4.09.1 package [45], and long terminal repeats (LTRs) were detected using LTR_finder v.1.0.7 [46]. Finally, these library files were merged and the repeat contents were identified by RepeatMasker v4.1.2.

The annotation of protein-coding genes was constructed using a combination of de novo, homology-based, and RNA-seq-based prediction. De novo gene structures were predicted based on the masked genome using AUGUSTUS v3.3.1 [47] and GLIMMERHMM v3.0.2 [48]. For sequence homology-based gene prediction, protein sequences from six organisms (*Oryza sativa*, *Arabidopsis thaliana*, *Helianthus annuus*, *Daucus carota*, and *Lactuca sativa*) were chosen to generate homology gene structures. For RNA-seq-based gene structure prediction, the clean RNA-seq reads were mapped against the genome assembly using Bowtie 2 v2.3.43 [49]. Gene structure prediction was conducted using TOPHAT2 v2.1.1 [50] and cufflinks v2.2.1 [51]. Finally, the nonredundant gene models were integrated by MAKER v3.00 [52]. Functional annotations were assigned according to the best match of the alignments to the NCBI (NR), SwissProt, TrEMBL, InterPro, the Kyoto Encyclopedia of Genes and Genomes (KEGG), Gene Ontology (GO), and Pfam nonredundant protein databases. Transcription factor annotations were performed using ITAK v1.7 [53].

### 4.7. Collinearity Analysis and Whole-Genome Duplication (WGD) Analysis

The homologous genes within each of *S. taccada* and *S. hainanensis*, as well as between each of *S. taccada*, *S. hainanensis* and *H. annuus*, were identified by all-vs-all BLASTP with a cutoff e-value of 10^−5^, identity ≥ 40%. Collinear blocks were detected by MCScanX [54] with default parameters. The genomic distribution of collinear blocks was visualized using Circos v0.69-9 [55]. Protein sequences in the collinear blocks were extracted and aligned using MAFFT v7.480, and codon sequences were obtained using PAL2NAL v14 [56]. Synonymous substitution rates (Ks) were calculated by KAKS_calculator v2.0 with the YN substitution model [57].

### 4.8. Phylogeny Construction and Divergence Time Estimation

To estimate divergence times, we used OrthoFinder v2.5.4 to identify orthologous gene groups for *S. taccada*, *S. hainanensis*, and nine other angiosperms species (*Helianthus annuus*, *Lactuca sativa*, *Daucus carota*, *Arctium lappa*, *Erigeron canadensis*, *Coffea canephora*, *Arabidopsis thaliana*, *Eutrema salsugineum*, and *Nelumbo nucifera*). All proteins from these eleven species were merged to perform an all-to-all alignment using BLASTP with a cutoff e-value of 1 × 10^−10^. Single-copy orthologs of the eleven species were aligned using MAFFT v7.480, and codon sequences were obtained using PAL2NAL v14. The alignments were trimmed using GBLOCKS v0.91b [58] using default parameters. We used JMODELTEST2 [59] to select an appropriate nucleotide-substitution model for reconstructing the phylogeny. To reconstruct the phylogenetic tree, we used PhyML v3.3 [60] with the best-fit model (GTR + I + G) and 1000 bootstrap replicates. Finally, the program MCMCTREE from the PAML4.8 package was employed to estimate divergence times, using “seq like (usedata = 1)”, “JC69 (model = 0, alpha = 0)”, and “independent rates (clock = 2)”. The root node of eudicots and monocots was constrained between 125–247 Mya and the common ancestor of eudicots was placed at 119.6–128.63 Mya based on two reliable fossil calibrations [61]. The MCMC analyses were run for 500,000 generations and sampled every 10 generations, using a burn-in of 200,000 iterations.

### 4.9. Gene Group Evolution and Positive Selection Detection

Gene copy number evolution was examined using CAFE v4.2.1 [62]. We obtained the counts of genes in each group from OrthoFinder v2.5.4. Gene families with more than 100 gene copies in a single species were removed from this analysis. Expansion or contraction of a gene group was identified as significant with a *p*-value < 0.01.

To detect genes under positive selection, orthologs were identified from the 11 species mentioned above. Orthologs were retained according to the following criteria: (1) orthologous groups with a single copy in each species or (2) low-copy orthologs that had only a single copy in the outgroup (*A. thaliana*) and less than five copies in all other species. The latter type of ortholog was further identified using the following method: first, we aligned the single-copy gene of the outgroup against all copies from other species with BLASTP; then, the best hit was used as the representative ortholog. The orthologs were aligned using MAFFT v7.480 and the aligned peptide sequences were transformed to nucleotide acid codon sequences using PAL2NAL v14. The codon alignments were used as input for CODEML in the PAML 4.8 package to detect genes under positive selection, with the branch of *S. taccada*, *S. hainanensis*, or their common ancestor as the foreground branch. The likelihood ratio test (LRT) *p*-values were computed using the χ^2^ statistic with a freedom degree of one. To remove false discoveries, the *p*-values were transformed to Q-values using Benjamini-Hochberg method [63] for multiple test correction (FDR < 0.05). Functional annotations of the positively-selected genes were obtained from the TAIR database. GO and KEGG enrichment analyses of expanded and species-private gene groups and genes under positive selection were performed using the ClusterProfiler v.4.0.2 package [64].

### 4.10. Analyses of the FAR1 Gene Family

The structural domains of *FAR1* (PF03101) were obtained from the Pfam database (http://www. ebi.ac.uk/Tools/hm, accessed on 4 March 2023). HMMER v3.3.2 software [65] was used to search *FAR1* domains based on the hidden Markov model (HMM) file (PF03101) with standard parameters (e-values < 10^−5^). Then, the obtained *FAR1* candidates were rechecked using the NCBI CDD (Conserved Domains) database (https://www.ncbi.nlm.nih.gov/cdd, accessed on 4 March 2023) and SMART (http://smart.embl-heidelberg.de/, accessed on 4 March 2023) to remove false positive findings. The sequences of *FAR1* genes in *A. thaliana* were downloaded from the PlantTFDB dataset. The conserved domain motifs of *FAR1* members were identified by MEME v4.12.0 [66], with the number of motifs set to 10, the minimum length of motifs set to 6, and the maximum length set to 100.

The FAR1 protein sequences of *S. taccada*, *S. hainanensis*, and *A. thaliana* were aligned using MAFFT v7.480. The phylogenetic tree was constructed using the maximum likelihood (ML) algorithm by IQ-TREE v2.2.0.3 [67] with 1000 bootstrap values. To determine the physical position of *FAR1* genes on chromosomes, all the *FAR1* genes were mapped to the corresponding reference genome using TBtools v1.068 [68]. Gene duplication events were analyzed by DupGen_finder [69] with default parameters, using *H. annuus* as the outgroup.

## Figures and Tables

**Table 1:** Assembly and annotation statistics.

	*S. taccada*	*S. hainanensis*
Assembly summary (Scaffold length ≥ 10 kb)		
Number of scaffolds	430	1014
Total length (bp)	1,011,548,119	1,141,139,833
GC content (%)	34.78%	32.71%
N50 (scaffolds) (bp)	124,591,045	143,667,403
N90 (scaffolds) (bp)	92,220,726	106,356,671
No. of scaffolds ≥ N50	4	4
No. of scaffolds ≥ N90	8	8
BUSCO evaluation of genome assembly		
Complete BUSCOs	1525 (94.49%)	1514 (93.80%)
Single-copy BUSCOs	1510 (93.56%)	1485 (92.01%)
Duplicated BUSCOs	15 (0.93%)	29 (1.80%)
Fragmented BUSCOs	40 (2.48%)	41 (2.54%)
Missing BUSCOs	49 (3.04%)	59 (3.66%)
BUSCO evaluation of predicted gene models		
Complete BUSCOs	1878 (88.5%)	1974 (93.1%)
Single-copy BUSCOs	1844 (86.9%)	1922 (90.6%)
Duplicated BUSCOs	34 (1.6%)	52 (2.5%)
Fragmented BUSCOs	78 (3.7%)	57 (2.7%)
Fragmented BUSCOs	165 (7.8%)	90 (4.2%)
Annotation summary		
Number of genes	34,560	34,033
Mean gene length (bp)	2949	2902
Mean exon length (bp)	218	228
Mean exon counts per gene	4.7	4.6

## Data Availability

The sequences reported in this study have been deposited with the National Genomics Data Center (NGDC), China National Center for Bioinformation. The genome sequences and annotations of *S. taccada* and *S. hainanensis* have been deposited in the Genome Warehouse (https://ngdc.cncb.ac.cn/gwh, accessed on 13 April 2023) in the NGDC database under the accession numbers GWHBCJW00000000 and GWHBCJX00000000, respectively. The RNA-seq data have been deposited in the Genome Sequence Archive (https://ngdc.cncb.ac.cn/gsa, accessed on 13 April 2023) in the NGDC under the accession numbers CRX248343, CRX248344, CRX248345, CRX248346, and CRX248347 with BioProject ID PRJCA005451. The genomic and RNA sequences have additionally been deposited with the National Center for Biotechnology Information (NCBI) under BioProject ID PRJNA817364.

## Supplementary Materials

The following supporting information can be downloaded at: https://www.mdpi.com/article/10.3390/ijms24087355/s1.

## References

[1] Carolin R.C. (1992). Brunoniaceae, Goodeniaceae. Flora of Australia.

[2] Howarth D.G., Gustafsson M.H.G., Baum D.A., Motley T.J. (2003). Phylogenetics of the Genus Scaevola (Goodeniaceae): Implication for Dispersal Patterns across the Pacific Basin and Colonization of the Hawaiian Islands. Am. J. Bot..

[3] Jabaily R.S., Shepherd K.A., Gardner A.G., Gustafsson M.H.G., Howarth D.G., Motley T.J. (2014). Historical Biogeography of the Predominantly Australian Plant Family Goodeniaceae. J. Biogeogr..

[4] Howarth D.G., Baum D.A. (2005). Genealogical Evidence of Homoploid Hybrid Speciation in an Adaptive Radiation of Scaevola (Goodeniaceae) in the Hawaiian Islands. Evolution.

[5] Castillo-Campos G., García-Franco J., Martínez L. (2021). First Record of Naturalization of Scaevola Taccada (Gaertn.) Roxb. (Goodeniaceae) in Southeastern Mexico. BIR.

[6] Emura N., Denda T., Sakai M., Ueda K. (2014). Dimorphism of the Seed-Dispersing Organ in a Pantropical Coastal Plant, Scaevola Taccada: Heterogeneous Population Structures across Islands. Ecol. Res..

[7] Goldstein G., Drake D.R., Alpha C., Melcher P., Heraux J., Azocar A. (1996). Growth and Photosynthetic Responses of Scaevola Sericea, a Hawaiian Coastal Shrub, to Substrate Salinity and Salty Spray. Int. J. Plant Sci..

[8] Hong D., Howarth D.G. (1983). Goodeniaceae. Flora of China.

[9] Huang T.-C. (1998). Goodeniaceae. Flora of Taiwan.

[10] Su M., He S., Li W., Wang S., Tan F., Huang Y. (2016). Systematic Position of a Rare Plant Species Scaevola Hainanensis Based on ITS Sequence Analysis. Acta Sci. Nat. Univ. Sunyatseni.

[11] Ho K.Y., Deng S.L., Chang Y.H., Tsai C.S., Kao M.F., Hsiao J.Y. (2005). Genetic Variation of the Endangered Scaevola Hainanensis (Goodeniaceae) in the Jiangjun Stream Mouth, Taiwan. Taiwan J. For. Sci..

[12] Huang X., Ouyang X., Yang P., Lau O.S., Li G., Li J., Chen H., Deng X.W. (2012). *Arabidopsis* FHY3 and HY5 Positively Mediate Induction of *COP1* Transcription in Response to Photomorphogenic UV-B Light. Plant Cell.

[13] Wang H., Wang H. (2015). Multifaceted Roles of FHY3 and FAR1 in Light Signaling and Beyond. Trends Plant Sci..

[14] Krzeszowiec W., Novokreshchenova M., Gabryś H. (2020). Chloroplasts in C3 Grasses Move in Response to Blue-Light. Plant Cell Rep..

[15] Huai J., Zhang X., Li J., Ma T., Zha P., Jing Y., Lin R. (2020). SEUSS and PIF4 Coordinately Regulate Light and Temperature Signaling Pathways to Control Plant Growth. Mol. Plant.

[16] Tang W., Wang W., Chen D., Ji Q., Jing Y., Wang H., Lin R. (2012). Transposase-Derived Proteins FHY3/FAR1 Interact with PHYTOCHROME-INTERACTING FACTOR1 to Regulate Chlorophyll Biosynthesis by Modulating *HEMB1* during Deetiolation in *Arabidopsis*. Plant Cell.

[17] Lin R., Wang H. (2004). Arabidopsis *FHY3/FAR1* Gene Family and Distinct Roles of Its Members in Light Control of Arabidopsis Development. Plant Physiol..

[18] Li G., Siddiqui H., Teng Y., Lin R., Wan X., Li J., Lau O.-S., Ouyang X., Dai M., Wan J. (2011). Coordinated Transcriptional Regulation Underlying the Circadian Clock in Arabidopsis. Nat. Cell Biol..

[19] Liu Y., Ma M., Li G., Yuan L., Xie Y., Wei H., Ma X., Li Q., Devlin P.F., Xu X. (2020). Transcription Factors FHY3 and FAR1 Regulate Light-Induced *CIRCADIAN CLOCK ASSOCIATED1* Gene Expression in Arabidopsis. Plant Cell.

[20] Li H., Handsaker B., Wysoker A., Fennell T., Ruan J., Homer N., Marth G., Abecasis G., Durbin R. (2009). 1000 Genome Project Data Processing Subgroup The Sequence Alignment/Map Format and SAMtools. Bioinformatics.

[21] Seppey M., Manni M., Zdobnov E.M. (2019). BUSCO: Assessing Genome Assembly and Annotation Completeness. Methods Mol. Biol..

[22] Camacho C., Coulouris G., Avagyan V., Ma N., Papadopoulos J., Bealer K., Madden T.L. (2009). BLAST+: Architecture and Applications. BMC Bioinform..

[23] Zhang C., Zhang T., Luebert F., Xiang Y., Huang C.-H., Hu Y., Rees M., Frohlich M.W., Qi J., Weigend M. (2020). Asterid Phylogenomics/Phylotranscriptomics Uncover Morphological Evolutionary Histories and Support Phylogenetic Placement for Numerous Whole-Genome Duplications. Mol. Biol. Evol..

[24] Bennetzen J.L. (2005). Mechanisms of Recent Genome Size Variation in Flowering Plants. Ann. Bot..

[25] Dai S., Zhu X., Hutang G., Li J., Tian J., Jiang X., Zhang D., Gao L. (2022). Genome Size Variation and Evolution Driven by Transposable Elements in the Genus Oryza. Front. Plant Sci..

[26] Liu J., Shi C., Shi C.-C., Li W., Zhang Q.-J., Zhang Y., Li K., Lu H.-F., Shi C., Zhu S.-T. (2020). The Chromosome-Based Rubber Tree Genome Provides New Insights into Spurge Genome Evolution and Rubber Biosynthesis. Mol. Plant.

[27] Albalat R., Cañestro C. (2016). Evolution by Gene Loss. Nat. Rev. Genet.

[28] Fang Y., Jiang J., Hou X., Guo J., Li X., Zhao D., Xie X. (2022). Plant Protein-Coding Gene Families: Their Origin and Evolution. Front. Plant Sci..

[29] Jin J., Tian F., Yang D.-C., Meng Y.-Q., Kong L., Luo J., Gao G. (2017). PlantTFDB 4.0: Toward a Central Hub for Transcription Factors and Regulatory Interactions in Plants. Nucleic Acids Res..

[30] Borges A., Rosa M.S., Recchia G.H., de Queiroz-Silva J.R., de Andrade Bressan E., Veasey E.A. (2009). CTAB Methods for DNA Extraction of Sweetpotato for Microsatellite Analysis. Sci. Agric..

[31] Yang G., Zhou R., Tang T., Shi S. (2008). Simple and Efficient Isolation of High-Quality Total RNA from *Hibiscus Tiliaceus*, a Mangrove Associate and Its Relatives. Prep. Biochem. Biotechnol..

[32] He Z., Feng X., Chen Q., Li L., Li S., Han K., Guo Z., Wang J., Liu M., Shi C. (2022). Evolution of Coastal Forests Based on a Full Set of Mangrove Genomes. Nat. Ecol. Evol..

[33] Chen Y., Chen Y., Shi C., Huang Z., Zhang Y., Li S., Li Y., Ye J., Yu C., Li Z. (2018). SOAPnuke: A MapReduce Acceleration-Supported Software for Integrated Quality Control and Preprocessing of High-Throughput Sequencing Data. GigaScience.

[34] Marçais G., Kingsford C. (2011). A Fast, Lock-Free Approach for Efficient Parallel Counting of Occurrences of *k*-Mers. Bioinformatics.

[35] Liu B., Shi Y., Yuan J., Hu X., Zhang H., Li N., Li Z., Chen Y., Mu D., Fan W. (2013). Estimation of Genomic Characteristics by Analyzing K-Mer Frequency in de Novo Genome Projects. arXiv.

[36] Weisenfeld N.I., Kumar V., Shah P., Church D.M., Jaffe D.B. (2017). Direct Determination of Diploid Genome Sequences. Genome Res..

[37] Xu M., Guo L., Gu S., Wang O., Zhang R., Peters B.A., Fan G., Liu X., Xu X., Deng L. (2020). TGS-GapCloser: A Fast and Accurate Gap Closer for Large Genomes with Low Coverage of Error-Prone Long Reads. GigaScience.

[38] Servant N., Varoquaux N., Lajoie B.R., Viara E., Chen C.-J., Vert J.-P., Heard E., Dekker J., Barillot E. (2015). HiC-Pro: An Optimized and Flexible Pipeline for Hi-C Data Processing. Genome Biol..

[39] Durand N.C., Shamim M.S., Machol I., Rao S.S.P., Huntley M.H., Lander E.S., Aiden E.L. (2016). Juicer Provides a One-Click System for Analyzing Loop-Resolution Hi-C Experiments. Cell Systems.

[40] Durand N.C., Robinson J.T., Shamim M.S., Machol I., Mesirov J.P., Lander E.S., Aiden E.L. (2016). Juicebox Provides a Visualization System for Hi-C Contact Maps with Unlimited Zoom. Cell Systems.

[41] Dudchenko O., Batra S.S., Omer A.D., Nyquist S.K., Hoeger M., Durand N.C., Shamim M.S., Machol I., Lander E.S., Aiden A.P. (2017). De Novo Assembly of the Aedes Aegypti Genome Using Hi-C Yields Chromosome-Length Scaffolds. Science.

[42] Jurka J., Kapitonov V.V., Pavlicek A., Klonowski P., Kohany O., Walichiewicz J. (2005). Repbase Update, a Database of Eukaryotic Repetitive Elements. Cytogenet. Genome Res..

[43] Tarailo-Graovac M., Chen N. (2009). Using RepeatMasker to Identify Repetitive Elements in Genomic Sequences. CP Bioinform..

[44] Abrusán G., Grundmann N., DeMester L., Makalowski W. (2009). TEclass—A Tool for Automated Classification of Unknown Eukaryotic Transposable Elements. Bioinformatics.

[45] Benson G. (1999). Tandem Repeats Finder: A Program to Analyze DNA Sequences. Nucleic Acids Res..

[46] Xu Z., Wang H. (2007). LTR-FINDER: An Efficient Tool for the Prediction of Full-Length LTR Retrotransposons. Nucleic Acids Res..

[47] Stanke M., Keller O., Gunduz I., Hayes A., Waack S., Morgenstern B. (2006). AUGUSTUS: Ab Initio Prediction of Alternative Transcripts. Nucleic Acids Res..

[48] Majoros W.H., Pertea M., Salzberg S.L. (2004). TigrScan and GlimmerHMM: Two Open Source Ab Initio Eukaryotic Gene-Finders. Bioinformatics.

[49] Langmead B., Salzberg S.L. (2012). Fast Gapped-Read Alignment with Bowtie 2. Nat Methods.

[50] Kim D., Pertea G., Trapnell C., Pimentel H., Kelley R., Salzberg S.L. (2013). TopHat2: Accurate Alignment of Transcriptomes in the Presence of Insertions, Deletions and Gene Fusions. Genome Biol..

[51] Trapnell C., Roberts A., Goff L., Pertea G., Kim D., Kelley D.R., Pimentel H., Salzberg S.L., Rinn J.L., Pachter L. (2012). Differential Gene and Transcript Expression Analysis of RNA-Seq Experiments with TopHat and Cufflinks. Nat. Protoc..

[52] Cantarel B.L., Korf I., Robb S.M.C., Parra G., Ross E., Moore B., Holt C., Sánchez Alvarado A., Yandell M. (2008). MAKER: An Easy-to-Use Annotation Pipeline Designed for Emerging Model Organism Genomes. Genome Res..

[53] Zheng Y., Jiao C., Sun H., Rosli H.G., Pombo M.A., Zhang P., Banf M., Dai X., Martin G.B., Giovannoni J.J. (2016). ITAK: A Program for Genome-Wide Prediction and Classification of Plant Transcription Factors, Transcriptional Regulators, and Protein Kinases. Mol. Plant.

[54] Wang Y., Tang H., Debarry J.D., Tan X., Li J., Wang X., Lee T.H., Jin H., Marler B., Guo H. (2012). MCScanX: A Toolkit for Detection and Evolutionary Analysis of Gene Synteny and Collinearity. Nucleic Acids Res..

[55] Krzywinski M., Schein J., Birol İ., Connors J., Gascoyne R., Horsman D., Jones S.J., Marra M.A. (2009). Circos: An Information Aesthetic for Comparative Genomics. Genome Res..

[56] Suyama M., Torrents D., Bork P. (2006). PAL2NAL: Robust Conversion of Protein Sequence Alignments into the Corresponding Codon Alignments. Nucleic Acids Res..

[57] Wang D., Zhang Y., Zhang Z., Zhu J., Yu J. (2010). KaKs_Calculator 2.0: A Toolkit Incorporating Gamma-Series Methods and Sliding Window Strategies. Genom. Proteom. Bioinform..

[58] Castresana J. (2000). Selection of Conserved Blocks from Multiple Alignments for Their Use in Phylogenetic Analysis. Mol. Biol. Evol..

[59] Darriba D., Taboada G.L., Doallo R., Posada D. (2012). JModelTest 2: More Models, New Heuristics and Parallel Computing. Nat. Methods.

[60] Guindon S., Dufayard J.-F., Lefort V., Anisimova M., Hordijk W., Gascuel O. (2010). New Algorithms and Methods to Estimate Maximum-Likelihood Phylogenies: Assessing the Performance of PhyML 3.0. Syst. Biol..

[61] Morris J.L., Puttick M.N., Clark J.W., Edwards D., Kenrick P., Pressel S., Wellman C.H., Yang Z., Schneider H., Donoghue P.C.J. (2018). The Timescale of Early Land Plant Evolution. Proc. Natl. Acad. Sci. USA.

[62] Han M.V., Thomas G.W.C., Lugo-Martinez J., Hahn M.W. (2013). Estimating Gene Gain and Loss Rates in the Presence of Error in Genome Assembly and Annotation Using CAFE 3. Mol. Biol. Evol..

[63] Benjamini Y., Hochberg Y. (1995). Controlling the False Discovery Rate: A Practical and Powerful Approach to Multiple Testing. J. R. Stat. Soc. Ser. B (Methodol.).

[64] Wu T., Hu E., Xu S., Chen M., Guo P., Dai Z., Feng T., Zhou L., Tang W., Zhan L. (2021). ClusterProfiler 4.0: A Universal Enrichment Tool for Interpreting Omics Data. Innovation.

[65] Mistry J., Finn R.D., Eddy S.R., Bateman A., Punta M. (2013). Challenges in Homology Search: HMMER3 and Convergent Evolution of Coiled-Coil Regions. Nucleic Acids Res..

[66] Bailey T.L., Johnson J., Grant C.E., Noble W.S. (2015). The MEME Suite. Nucleic Acids Res..

[67] Minh B.Q., Schmidt H.A., Chernomor O., Schrempf D., Woodhams M.D., von Haeseler A., Lanfear R. (2020). IQ-TREE 2: New Models and Efficient Methods for Phylogenetic Inference in the Genomic Era. Mol. Biol. Evol..

[68] Chen C., Chen H., Zhang Y., Thomas H.R., Frank M.H., He Y., Xia R. (2020). TBtools: An Integrative Toolkit Developed for Interactive Analyses of Big Biological Data. Mol. Plant.

[69] Qiao X., Li Q., Yin H., Qi K., Li L., Wang R., Zhang S., Paterson A.H. (2019). Gene Duplication and Evolution in Recurring Polyploidization–Diploidization Cycles in Plants. Genome Biol..

